# Ecophysiological and behavioural response of juveniles of the Chilean cold-water coral *Caryophyllia (Caryophyllia) huinayensis* to increasing sediment loads

**DOI:** 10.1038/s41598-023-47116-6

**Published:** 2023-12-06

**Authors:** Melanie Fähse, Covadonga Orejas, Jürgen Titschack, Günter Försterra, Claudio Richter, Jürgen Laudien

**Affiliations:** 1 Alfred-Wegener-Institut Helmholtz-Zentrum für Polar- und Meeresforschung, 27515 Bremerhaven, Germany; 2 Instituto Español de Oceanografía, Centro Oceanográfico de Gijón (IEO, CSIC), Avenida Príncipe de Asturias 70 Bis, 33212 Gijón, Spain; 3 Hanse-Wissenschaftskolleg – Institute for Advanced Study (HWK), Lehmkuhlenbusch 4, 27753 Delmenhorst, Germany; 4 MARUM-Center for Marine Environmental Sciences, University of Bremen, Leobener Str. 8, 28359 Bremen, Germany; 5 Senckenberg Am Meer, Marine Research Department, Südstrand 40, 26382 Wilhelmshaven, Germany; 6 Huinay Scientific Field Station, Casilla 462, Puerto Montt, Chile; 7 Facultad de Recursos Naturales, Pontificia Universidad Católica de Valparaíso, Escuela de Ciencias del Mar, Valparaíso, Chile; 8 Universität Bremen, Bibliothekstraße 1, 28359 Bremen, Germany

**Keywords:** Ecology, Zoology, Ecology, Ocean sciences

## Abstract

Chilean Patagonia is a hotspot of biodiversity, harbouring cold-water corals (CWCs) that populate steep walls and overhangs of fjords and channels. Through anthropogenic activities such as deforestation, roadworks, aquafarming and increased landslide frequency, sediment input increases in the fjord region. While the absence of CWCs on moderately steep slopes has been suggested to reflect high vulnerability to sedimentation, experimental evidence has been lacking. Here, we investigated the sensitivity of CWCs to sediment stress, using juvenile *Caryophyllia (Caryophyllia) huinayensis* as a model. A 12-week aquarium experiment was conducted with three sediment loads: the average natural sediment concentration in Comau Fjord, 100- and 1000-fold higher sediment levels, expected from gravel road use and coastal erosion. Changes in coral mass and calyx dimensions, polyp expansion, tissue retraction and respiration were measured. For CWCs exposed to two and three order of magnitude higher sediment concentrations, 32% and 80% of the animals experienced a decrease in tissue cover, respectively, along with a decrease in respiration rate of 34% and 66%. Under the highest concentration corals showed reduced polyp expansion and a significantly reduced growth of ~ 95% compared to corals at natural concentration. The results show that *C.* *huinayensis* is affected by high sediment loads. As human activities that increase sedimentation steadily intensify, coastal planners need to consider detrimental effects on CWCs.

## Introduction

Cold-water corals (CWCs) are found in all oceans from the intertidal zone to abyssal depths^1–3^. They form three-dimensional habitats, providing niches and nurseries for a variety of species^2,4–6^. However, anthropogenic changes are challenging CWCs’ capability to acclimate and adapt at local, regional and global scales. One important stressor at the local level is the increasing sediment input to coastal habitats due to higher terrestrial runoff, along with changes in the catchment area (e.g., agriculture, deforestation), on the coast (e.g., construction of buildings and roads) and at sea (e.g., drilling, bottom trawling)^7–10^.

Over the last two decades, Chilean Patagonia has undergone a profound change from a remote, pristine habitat to the world’s second largest producer of farmed salmon^11^. The spread of salmon farms in the fjord region^12,13^ has led to an increase in organic and inorganic sediment loads due to direct and indirect effects, directly, due to eutrophication (excess feed, waste, faeces, nutrients triggering harmful blooms) and indirectly, due to supporting infrastructure (e.g. roadworks)^12,14–18^. Sedimentation rates in the vicinity of Chilean salmon farms have not been documented yet, but Kutti et al.^19^ recorded nine times higher sedimentation rates in a radius of 250 m around a salmon farm in a Norwegian fjord^15,20^. An imminent threat to the corals in the Chilean fjord region is the planned road construction of the ‘Carretera Austral’ along the steep slopes of the fjords. The road is planned to pass through forested escarpments, for which parts of the rock walls will have to be blasted. This will lead to a high input of rock material and sediments into the fjords, and later also to increased sedimentation from the unpaved road, as documented for other areas^21^. In addition, deforestation and the associated fragmentation or removal of root systems reduce the stability of the soil. This increases the risk of landslides in these steep areas with low organic cover^13,22–24^. Further, increased precipitation and glacier melt due to climate change locally enhance terrestrial material input^17,25,26^.

Sedimentation is well known to affect the performance of warm water corals (WWCs) (i.e.^8,27–30^). Similar effects of sediment loads have been observed in CWCs, but the information is so far limited to the branching scleractinian *Desmophyllum pertusum* (syn. *Lophelia pertusa*^31^)^7,32–36^ and studies have mostly focused on the effect of sediments produced and used in drilling operations^7,32,34,35^, while terrigenous sediment sources are neglected. However, the effects of higher sedimentation result in alterations in polyp behaviour, mucus release and respiration rates in corals^9,28,30,32,37,38^. Consequently, they have less energy available to grow and/or reproduce^9,28,30^. The extreme case occurs when corals are covered by sediment, as in this situation they may suffocate and die^8,36,39^. In response, some corals change their morphology and there may be variations in the composition of the associated community^30,40^. The effect of sediment on coral performance depends not only on its quantity, but also on its characteristics (e.g., grain size, type, and organic content) and the duration of sediment exposure^36,39,41^. For instance, fine resuspended materials can clog the feeding mechanism^34,40,42^. However, some coral species, such as the CWC *D.* *pertusum,* seem to tolerate natural variations in sediment input^35^, but mortality increases with prolonged or high sediment loads^33^. Nevertheless, the stress caused by increasing sediment loads has different effects on individual species, as well as on different stages of their life cycles^39,40^. For example, Jones et al.^28^ indicated that the survival and settlement of WWC larvae is negatively affected under sedimentation. Moeller et al.^9^ demonstrated for the tropical coral species *Leptastrea purpurea* that the tolerance threshold of adults is at least one order of magnitude higher than that of recruits. Similarly, Fabricius et al.^43^ found up to two orders of magnitude higher sensitivity of juvenile compared to adult corals. Although juveniles are paramount for the survival of the species^28,34^, there are very few studies on the effects of sediment loads (and stressors of anthropogenic origin in general) on juvenile corals^9,44^ and none for CWCs. To fill this knowledge gap, it is important to consider the early life stages of corals, which is what we aimed for with this study.

**Figure 1:**
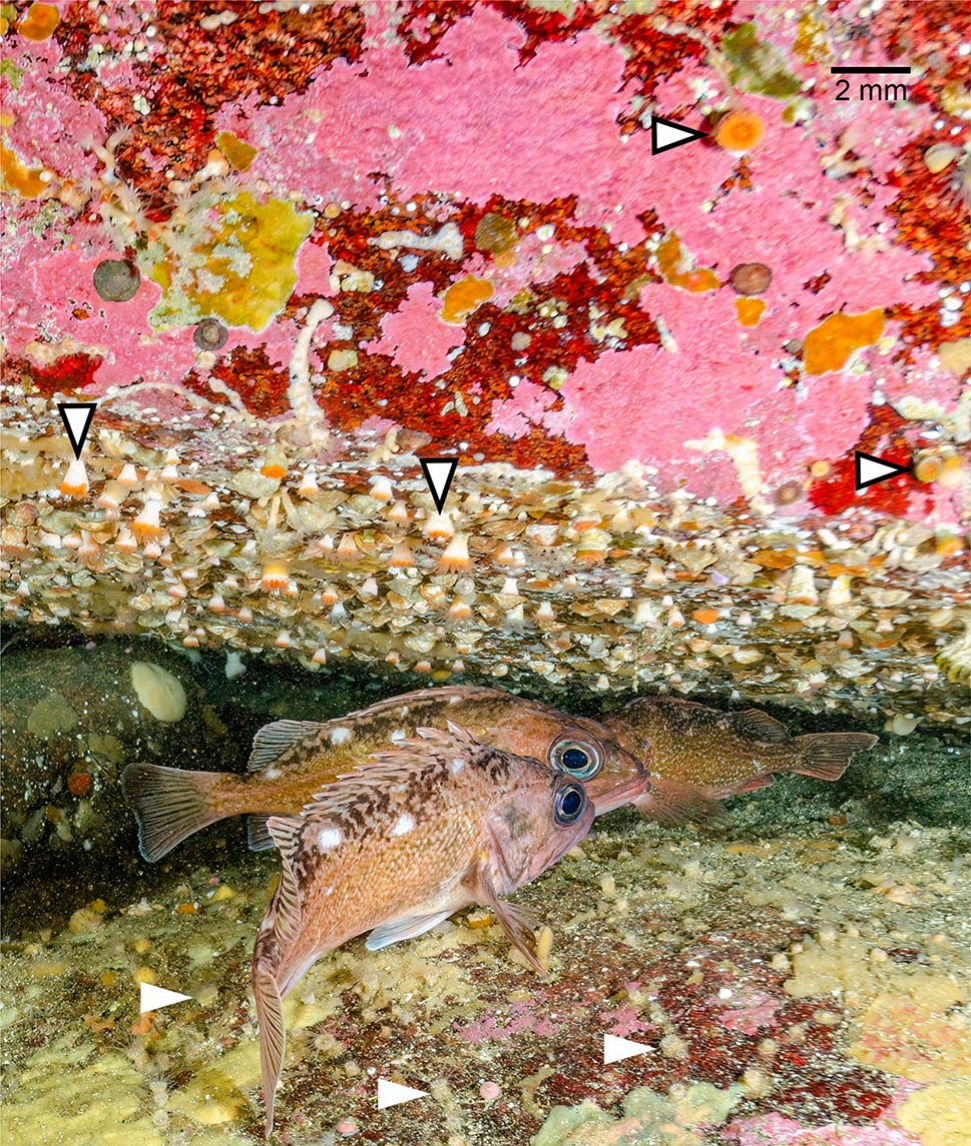
Downwards and sideways oriented *C. huinayensis* individuals (black-outlined arrows) on overhang in Comau Fjord providing shelter to Patagonian redfish (*Sebastes oculatus*); Note: there are no upward facing corals, all animals facing upwards (white arrows) are encrusting anemones *(Epizoanthus fiordicus, Zoantharia)*. Photo credit: Thomas Heran, AWI.

Chilean Comau Fjord (approx. 42° 29′ S–42° 11′ and 72° 22′ E–72° 35′) is a region characterized by high precipitation rates, high river run-off and natural landslides contributing to high sediment input^22^. Here, CWCs thrive with the greatest abundance on steep rocky slopes, vertical walls and overhangs providing putative shelter from sediment stress^12,22^. In Comau Fjord *Caryophyllia (Caryophyllia) huinayensis* Cairns, Häussermann & Försterra, 2005^45^, an azooxanthellate, solitary Scleractinian CWC, endemic to Chilean waters^45^, grows laterally on vertical walls or downwards beneath overhangs, colonizing bedrock, but also biogenic hard substratum such as living molluscs^45^. The species is frequently found in association with two of the three other scleractinian CWC species—*Desmophyllum dianthus* and *Tethocyathus endesa*^12,16,45^. While *T.* *endesa* dominates the uppermost part of the overhang, *C.* *huinayensis* is usually found on the almost vertical walls and *D. dianthus* below. Large schools of scorpaenid Patagonian redfish (*Sebastes* *oculatus,* mistakenly identified as *S. capensis*^45^) are found around *C.* *huinayensis* and *D. dianthus* aggregations^45^. The fish may clean the corals of sediment through their movements due to a sweeping effect (Fig. 1). This natural occurrence of *C.* *huinayensis* in habitats sheltered from sedimentation may indicate its sensibility to sediment stress.

To understand and predict the effects of increased sedimentation on the physiological performance and behaviour of juvenile *C.* *huinayensis*, we investigated the effects of natural (C, 0.0042 mL L^−1^), 100-fold (T1, simulating heavily used gravel roads^21^) and 1000-fold (T2, simulating coastal erosion and landslide^46^) higher sediment concentrations using a 12-week *ex-situ* experiment. Corals were placed sideways and downwards to mimic natural growth directions in Comau Fjord, when exposed to experimental sediment concentrations. We measured juvenile growth, polyp behaviour, tissue cover changes and respiration rate. We expected a deleterious effect on the physiological performance with increasing sediment load, and therefore slower growth rates and higher respiration rates. Further we suspected a reduced polyp expansion as a protection mechanism against clogging and erosion caused by high sediment load and retraction of coral tissue in response to stress of the coral juveniles.

## Results

### Coral mortality

No mortality of the juvenile specimens of *C. huinayensis* was observed in any of the treatments throughout the experiment.

### Coral growth

**Figure 2:**
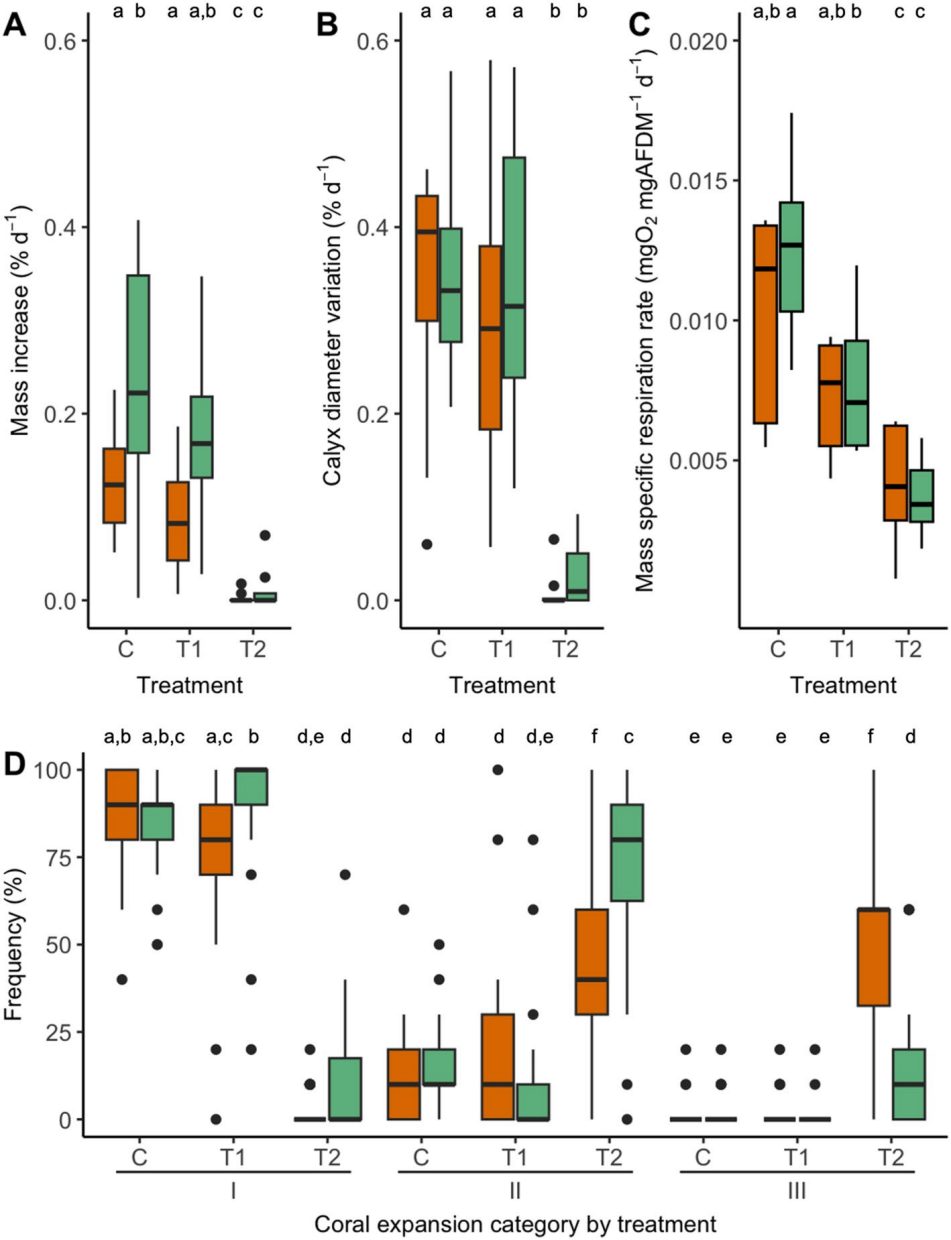
Mass (**A**) and calyx diameter (**B**) variation, mass-specific respiration rates (**C**) and polyp expansion (**D**) of juvenile *C. huinayensis* exposed to C, T1, T2 conditions. Orange boxes display growth of sideways oriented and green boxes of downward oriented corals. The box represents the interquartile range of values, the black horizontal line the median, the whiskers the standard deviations and the black dots the outliers. The significance levels between treatments are indicated as letters, with different letters representing significant differences. In (**D**) significance letters refer only to the treatments within the category. No differences in growth rate were observed between the intermediate measurement (after 6 weeks) and the final measurement (after 12 weeks).

Calyx growth (mass increase) of *C.* *huinayensis* did not differ between the first half of the experiment (week 0–6) and the second half (week 7–12). However, the growth rate differed between treatments (SRH-test, *p* < 0.001). Corals under T2 (n = 19) had decreased growth rates (Kruskal–Wallis test + post-hoc Dunn-Bonferroni: *p* < 0.001) of 96%, while corals under T1 (n = 19) had no reduction in growth compared to specimens exposed to the natural sediment concentration (C, n = 20) (Fig. 2A). Overall, the sideways oriented corals grew slower than downward oriented ones (SRH-Test, *p* < 0.05; Supp. Mat. 6) (Fig. 2A).

The increase in calyx diameter of corals was 95% lower under T2 conditions (n = 20; Kruskal–Wallis, post-hoc Dunn-Bonferroni: *p* < 0.001) than for the corals under C conditions (n = 20; Fig. 2B), while T1 (n = 20) calyx diameter did not differ from that of C. In contrast to the buoyant weight, no differences in calyx diameter growth were detected between sideways and downward oriented specimens (SRH-Test, *p* = 0.7; Supp. Mat. 6) (Fig. 2B).

### Oxygen consumption

After 13 weeks, the mass-specific respiration rate of *C.* *huinayensis* differed among treatments (ANOVA, *p* < 0.001; Supp. Mat. 6). Overall oxygen consumption decreased with increasing sediment concentration. The lowest respiration rates were measured in corals under T2 (C: 11.3 ± 3.8 µg O_2_ mg ash-free dry mass (AFDM)^−1^ d^−1^ (n = 10); T1: 7.5 ± 2.4 µg O_2_ mg AFDM^−1^ d^−1^ (n = 10), i.e. reduced by 34% compared to the respiration of corals under C conditions; T2: 3.9 ± 1.9 µg O_2_ mg AFDM^−1^ d^−1^ (n = 10) i.e. reduced by 66% compared to the respiration of corals under C conditions) (Fig. 2C). Respiration rates of sideways and downward orientated corals did not differ between treatments (two-way ANOVA, *p* > 0.05; Supp. Mat. 6).

### Polyp expansion

Under C (n = 20) and T1 (n = 20) most of the corals (C: 85.25 ± 12.22%, T1: 85.73 ± 19.57%) showed fully polyp expansion (Category (Cat.) I) over the experimental period, while under T2 (n = 20) partly (Cat. II, 59.64 ± 28.93) or no expansion was (Cat. III, 32.38 ± 26.28) identified (Fig. 2D). All expansion categories differed among treatments (SRH: < 0.001, Supp. Mat. 6). Under T2 downward orientated individuals were more partly (Cat. II) and less fully retracted (Cat. III) than sideways orientated individuals (Tukey HSD: *p* < 0.05), where almost 50% of the individuals were fully retracted over the experimental period (Fig. 2D; Supp. Mat. 6).

### Tissue retraction

**Figure 3:**
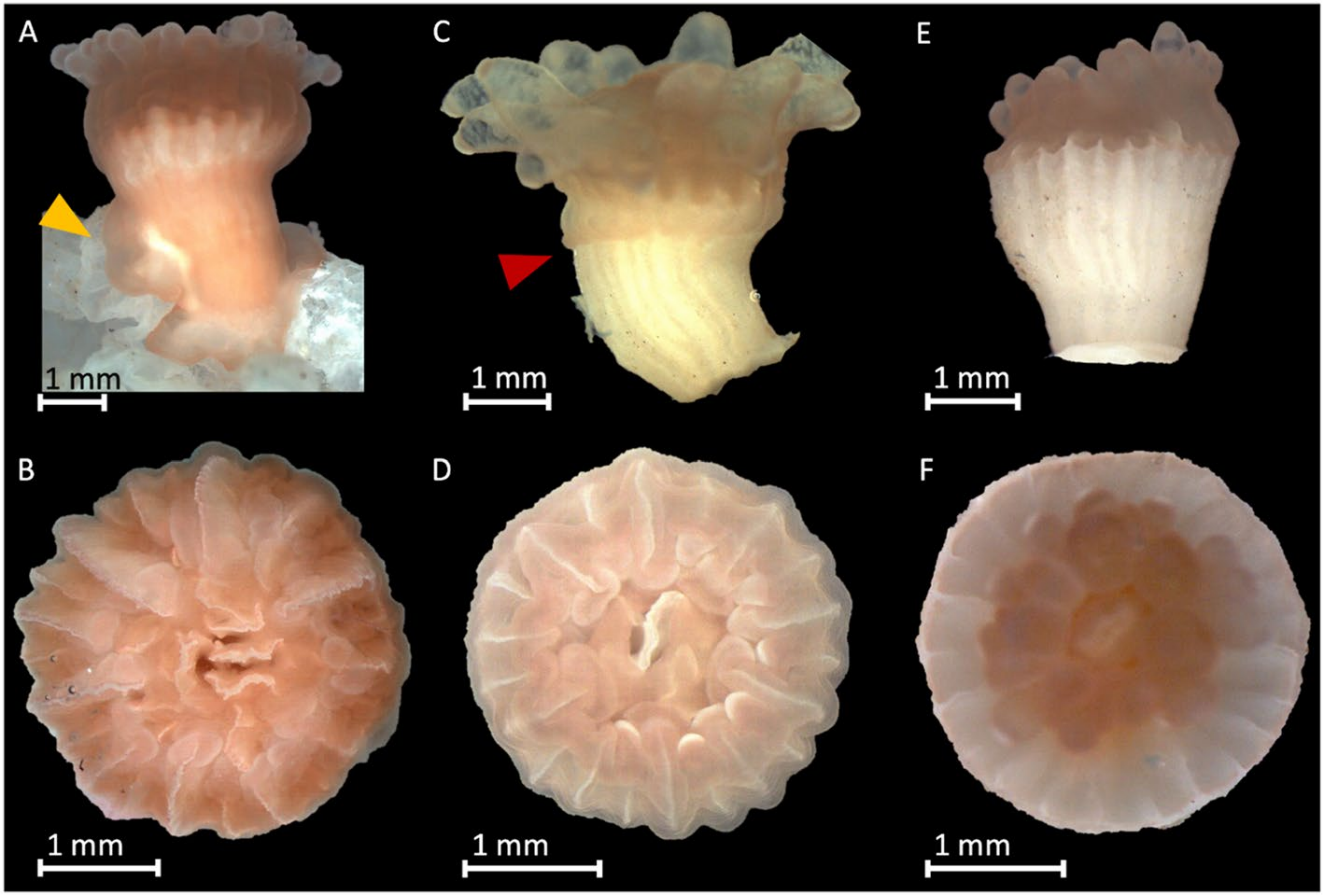
Tissue retraction in juvenile *C. huinayensis.* Photographs with differing tissue extension, (**A**, **B**) show corals after twelve weeks maintenance under control conditions; (**C**, **D**) corals after 6, and (**E**, **F**) after 12 weeks, under T2, respectively, showing retracted tissue. Yellow arrow indicates that the tissue is expanding over the substrate, red arrow indicates the edge where the tissue retracts from the skeleton.

Tissue retraction differed among the treatments (SRH: *p* < 0.001, Table 1). No tissue retraction was observed under C conditions (n = 20; Fig. 3A,B); some animals even expanded their tissue over the substrate of attachment (Fig. 3A). Under T1 (n = 20) 32 ± 48% of the corals partially retracted their tissue. A higher number of corals (Kruskal–Wallis + post-hoc Dunn-Bonferroni: *p* < 0.01) retracted tissue from the skeleton in T2 (80 ± 41%, n = 20) (Fig. 3C–F). Although not significant (SRH-Test: *p* > 0.05; Table 1), sideways oriented corals tended to retract their tissue more often than downward oriented ones.

**Table 1:** Mean percentage (± SD) of corals that retracted their tissue at the end of the experiment and results of the statistical analysis indicating the variables tested (T = Treatment, O = Orientation).

Orientation	C (%)	T1 (%)	T2 (%)	Significance test			
				Scheirer-Ray-Hare			
				Variable	df	H value	*p* value
Overall (n = 20)	0	32 ± 48	80 ± 41	T	2	27.2892	**< 0.001**
				O	1	0.968	0.325
				T:O	2	103.9	0.778
					Residuals: 53		

				Kruskal–Wallis			
				Variable	df	chi-squared	*p* value
Sideways (n = 10)	0	40 ± 51	90 ± 32	T	2	16.009	**< 0.001**
Downwards (n = 10)	0	27 ± 45	70 ± 46	T	2	11.509	**0.003**

Significance differences are marked in bold.

### Further observations

**Figure 4:**
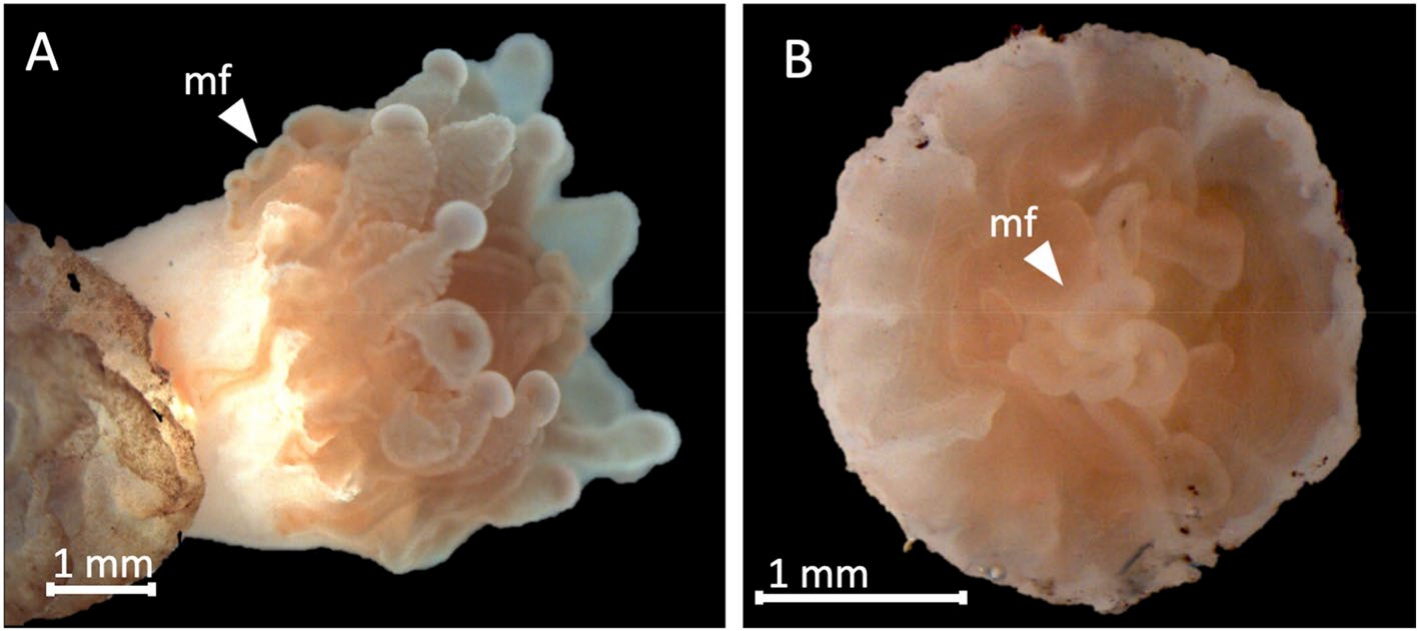
Two individuals of *C. huinayensis* kept under T2 extruding mesenterial filaments (mf).

One coral under T1 was found to produce mucus (Supp. Mat. 3) and under T2 some corals showed extruded mesenterial filaments (Fig. 4).

## Discussion

The results of this 12-week experiment reveal a non-linear response of juvenile *C. huinayensis* to sediment loads. Exposure experiments showed that the CWCs were able to tolerate sediment concentrations exceeding the average by two orders of magnitude. However, at sediment concentrations 1000-fold higher than ambient (T2), we found negative, but non-lethal effects on coral performance, including reduced growth, decreased polyp extension, respiration rate and tissue cover. This indicates that juvenile *C. huinayensis* are resilient for a medium-term period of 12 weeks to sediment loads simulating terrigenous inputs after heavy river run-off in natural catchment areas, but vulnerable to concentrations simulating an anthropogenically altered catchment area with soil erosion.

Based on buoyant weight and calyx diameter, we found no growth differences in corals maintained under ambient (C) and T1 conditions, but arrested growth under T2 condition (Fig. 2). Growth rates under the former were at the higher end of published values for CWC (Table SM1). As coral growth requires energy, the biological mechanisms governing the growth responses are related to energy availability and allocation^47^. High sediment loads may impair the capture efficiency of suspension feeders on plankton prey and thus energy uptake and availability^48^. Severe turbidity also leads to cleaning responses such as mucus production, ciliary motion and tentacle movements to prevent smothering by sediments settling on the coral surface^30^. This may shift assimilated energy away from growth. The lack of a negative growth response under T1 suggests that energy availability outweighs changes in energy allocation, even at 100-fold higher than natural sediment loads. This may be due to sufficient food supply and uptake and ample oxygen allowing aerobic energy production and/or energy reserves (e.g. storage lipids, cf.^49^) in the coral tissue sustaining growth. In the Mediterranean CWC *Madrepora oculata* only a small portion (1–3%) of the total metabolic energy is required for calcification and therefore the corals can maintain their calcification and thus their growth even under unfavourable conditions^50^. In addition, abundant feeding may help animals to compensate for stress^51,52^. Indeed, under low seawater pH, well-fed *C. huinayensis* juveniles increased their growth rates compared to those kept under low feeding conditions^51^. Similarly, Martínez-Dios et al.^52^ detected a positive effect on net calcification rate and overall performance at a higher feeding frequency in *D. dianthus* kept in different pH environments.

The pronounced growth decline of *C. huinayensis* under T2 (~ 95% lower growth rate relative to the one under C conditions as assessed by buoyant weight and calyx diameter) thus probably indicates a strong change in energy uptake and/or allocation. It is thus possible that a threshold concentration of suspended sediments was exceeded triggering an interruption of the feeding and/or cleaning response. To rule out that the increased phosphate values measured in T2 cause lower growth rates in the corals, we tested coral growth in an experiment where phosphate values without turbidity were increased. We found that increased turbidity indeed is more decisive for condition and growth of *C.* *huinayensis* juveniles than the possible influence by the phosphate originating from the sediment suspension (Supp. Mat. 2). Similarly, the physiological performance of the CWC *D. pertusum* seems to depend less on changes in seawater chemistry than on food availability^53^.

The sediment concentrations impacting coral growth vary widely in the literature and are associated with different coral species, sediment types and form of exposure^47^. In this regard, Larsson and Purser^36^ found that the addition of natural sediment and drill cuttings (65 and 195 mg cm^−2^, grain size < 1 mm) to aquaria with *D.* *pertusum* had no effect on their growth rate, but some polyps were covered in drill cuttings and consequently suffocated (Table 2). However, Larsson et al.^35^ showed reduced growth rates (50%) when the same species were exposed to drill cuttings and natural sediment suspension (25 mg L^−1^; Table 2) for 12 weeks, as was the case in our experiment. In fact, physiological changes in corals were up to 10 times accelerated under suspended sediments than in deposited sediments, while the opposite was true for tissue mortality^47^. These authors also suspected that the shift of energy resources to cleaning reduced or impaired food uptake. A study on the impact of macro- and microplastic particles in the water column on *D.* *pertusum* also evidenced reduced growth rates after 69 days^54^. The authors suggested that the plastic particles alter the hydrodynamic conditions around the coral and reduce the encounter rate between prey and polyp, thus affecting energy acquisition. Considering the results of our experiment, sediment particles could play a role similar to microplastics and reduce the effectiveness of food acquisition for the corals.

**Table 2:** Overview on responses to increased sedimentation on scleractinian CWCs and juvenile WWCs; w = weeks, d = days, h = hours.

Species	Sediment characteristics and sediment treatment	Exposure time and application	Experimental outcome	Source
*C. huinayensis* (CWC) Juveniles	Wadden Sea benthic sediment (Spieka-Neufeld) Freeze dried and boiled with H_2_0_2_ 1.6 (C)/160 (T1)/1600 mg L^−1^ (T2) Grain size < 200 µm	12 w Suspended sediment	· Under T2 decreased growth rate, polyp extension, tissue retraction · Decreased metabolic rate with increasing sediment load · Evidence of mucus production, mesenterial filaments extruded	This study
*D. pertusum* (CWC) Nubbins with adults	Bentonite (BE), Barite (BA), Drill cuttings (DC) ~4–~60 mg L^−1^	5 d, 4 h repeated pulses Effect measured after 2 and 6 w	· No significant differences in growth rate or respiration · Increase mucus production when BE-exposed · Polyp mortality increased significantly at DC doses · Indicated threshold ~20 mg L^-1^	Baussant et al.^7^
*D. pertusum* (CWC) Larvae (8 and 21 d)	BE, BA, DC 0/5/10/50/100/500 mg L^−1^ Grain size ≤ 63 µm	24 h Suspended sediment	· Sensitivity varies with exposed material (LC_50_ ~ 50 – 380 mg L^-1^) · Greatest sensitivity and lowest recovery potential under BE · Age-related effects which differed among test material	Järnegren et al.^34^
*Leptastrea purpurea* (WWC), Newly settled juveniles and adults; *Acropora hyacinthus* (WWC) newly settled juveniles	Benthic sediment (Family Beach, Apra Harbour) *A. hyacinthus* juveniles: 0/8.3/16.6 mg cm^−2^ d^−1^ *L. purpurea* juveniles: 0/16.6/33.3 mg cm^−2^ d^−1^ *L. purpurea* adults: 0/33.3/333.3 mg cm^−2^ d^−1^ Grain size 2–630 µm	3–5 w Suspended sediment	· Growth and survival of newly settled juveniles negatively affected, but not juveniles older than one month · No significant difference in mortality for adults under all concentrations	Moeller et al.^9^
*D. pertusum* (CWC)	Natural benthic sediment (Tisler Reef) DC, Mixture of DC and natural sediment 66/198/462 mg cm^−2^ Grain size < 1 mm	24, 48 and 72 h Poured evenly into the standing water as a homogeneous suspension and allowed to settle for 12 h before restarting the flow	· Decrease of oxygen concentration on coral surface by coverage with all three sediment types · Natural sediment higher effect in inducing anoxia than DC · Exposure to anoxic sediment, mucus release and withdrawn tentacles, exposure > 24 h lead to death of polyps	Allers et al.^32^
*D. pertusum* (CWC)	Natural benthic sediment (*D.* *pertusum* reef at Säcken, northern Koster-Fjord) Water-based DC 0/5/25 mg L^−1^ Grain size < 63 µm	12 w Suspended sediment	· Lower growth rates (50%) under high exposure · Trend: DC have a higher impact on growth than natural benthic sediment · Polyp extension less under high sediment concentration · No effect on respiration, proportions of tissue and fatty acids. No significant effect of additional mucus release on coral energy expenditure	Larsson et al.^35^
*D. pertusum* (CWC)	Natural benthic sediment (*D.* *pertusum* reef at Säcken, northern Koster-Fjord) Sediment rejection: 66/196 mg dry mass (DM) cm^−2^ Repeated exposure: 33 mg DM cm^−2^ Drilling Event/Burial study: 65/195 mg DM cm^−2^ Grain size < 1 mm Water-based DC Grain size < 1 mm and < 63 mm	12 h Sediment rejection experiment 45 d repeated exposure 3 w mimicked drilling-event Sediment allowed to settle for 6–12 h without water flow	· Tissue-covered surfaces are efficiently cleaned · Equal ability to reject different sediment types with same grain size · Starved and fed corals have the same ability to reject sediment · Sediment accumulated on tissue free skeleton, with repeated exposure, the sediment accumulated to the extent that it also spread to the adjacent regions of the skeleton covered with coenosarc, leading in some cases to smothering of the tissue and death · “Drilling event”: Number of coral fragments with smothered tissue and polyp mortality increased	Larsson and Purser^36^
*D. pertusum* (CWC)	Natural benthic sediment (Green Canyon lease block 354, Gulf of Mexico) Autoclaved 0/50/150/250/350 mg L^−1^ Grain size < 5 mm	14 d Suspended sediment 7 d, complete burial under sediment > 1 cm 10 h, sediment gently deposited on calyx surface with glass pipette until poured over the side	· Polyp survival decreased with increasing concentration · Critical survival between 2 and 4 d · Sediment was removed from calyx after 9 h	Brooke et al.^33^

Moeller et al.^9^ studied the effect of sediment on juvenile WWCs and documented the highest influence on growth (measured visually by comparing pictures of polyps) and survival in the first 4 weeks after settling. In the present study, no effect on calyx growth of *C.* *huinayensis* was detected between the first half of the experiment (week 0–6) and the second half (week 7–12). This suggests that the effect of sedimentation on juveniles of this CWC is constant after settlement and acclimatization, but also suggests that newly settled recruits may have a lower threshold towards sedimentation. However, the sediment concentration of T2 is higher than concentrations used in other studies (see Table 2), and therefore survival points to a naturally high resilience of the studied coral to sediment load. Since sedimentation can influence growth and thus alter morphology^30,40^, for example by causing corals to grow longer and less wide to avoid cover when sedimentation rates are high, it would be useful to additionally record linear growth and document other morphological changes in future studies.

While the differential growth responses under T1 and T2 suggested changes in energy availability and allocation (e.g., less energy for growth, more energy for cleaning), the respiration measurements showed a decline in metabolic rates with increasing sediment load (Fig. 2C). The lower oxygen consumption reflects a metabolic decline and therefore suggests a decline in energy availability, e.g., due to reduced food capture and/or processing with increasing sediment load. It could also be due to changes in the allocation of available energy for respiration, tissue growth, reproduction, production of particulate and dissolved organic matter and calcification^55^. Through respiration, corals produce Adenosine-5′-triphosphate (ATP), which is required for energy-consuming processes such as calcification, tissue growth or active sediment removal such as mucus production^9,30,56^. Our results contrast with the assumption that corals increase their respiration rate with increasing sediment load, as they need additional energy to expel the sediment^40^. Known influences on the respiration rate of the animals, such as temperature and feeding^57^, can be ruled out, as the experiment was conducted simultaneously at the same temperature and feeding regime for all treatments. In addition, no effect of feeding on the respiration of juvenile *C.* *huinayensis* has been detected after 24 h of starvation (unpublished data, Kristina Beck). Therefore, it is very likely that the reduced respiration rate of *C.* *huinayensis* results in a reduced metabolism and thus subsequent a lower growth rate. This has been observed in other coral species, e.g. the WWC *Pocillopora acuta*, whose metabolic rate decreased when exposed to high sediment and nutrient load; calcification and coral cover also decreased^58^. Naumann et al.^59^ also found reduced respiration with a simultaneous reduction in calcification for *D. dianthus* when not fed for 3 weeks. This indicates that the juvenile *C.* *huinayensis* may have consumed less food (despite the same availability) under T1 and T2 due to the increase in sediment load over time. This might have resulted in an energy deficit in the animals and a slowdown in respiration. The animals under T1 were still able to put the necessary energy into growth, while the animals under T2 reduced their metabolism to the point where growth was negligible. As for the effects of other stressors on respiration rates, Hennige et al.^56^ documented decreased respiration in *D.* *pertusum* under increased carbon dioxide (CO_2_) concentrations, but no change in calcification, which indicates an energetic imbalance. However, respiration rate and calcification decreased in *D. dianthus* when exposed to multiple stressors such as elevated temperature and elevated partial pressure of carbon dioxide (pCO_2_)^60^. Deep-sea corals (Caryophyllidae, *Dendrophyllia* sp., *Eguchipsammia* *fistula*) from the Red Sea may be adapted to oxygen-limited, highly oligotrophic and 20 °C warm waters with low respiration and calcification rates and keep their tissue alive at the tips of their skeletons to minimize the metabolic needs^61^. Allers et al.^32^ revealed that settling sediment may reduce respiration due to limited oxygen accessibility to the CWC *D. pertusum* when it was covered under anoxic sediment for > 24 h. Weber et al.^62^ found tissue degradation after 1 d when the tropical coral *Montipora peltiformis* was exposed to organic-rich sediment, while organic-poor sediment had no effect after 6 d and postulated, that this was microbially mediated. However, in our experiment, the sediment was organic free, not anoxic and we did not see any sediment accumulation on the coral surface thus a limited exchange of gases, both the output of CO_2_ and the input of oxygen is unlikely.

Regarding polyp expansion, most of the corals under T2 were either fully retracted or showed partly expansion while corals under T1 and C conditions appeared primarily fully expanded. The retraction of the tentacles may protect the coral from the deleterious effects of sedimentation, as finer particles may be harder to repel and could clog the feeding mechanism^30,40^. Some WWCs ingest sediment particles under turbid conditions and can derive nutritional value from them, while most corals cease activity when confronted with heavy sediment loads^40^. Therefore, the juveniles may save energy by retracting the tentacles, as the sediment does not contain food due to H_2_O_2_ treatment, hence not profitable for the animals. In addition, the sediment may have a high erosion potential and retracting the tentacles may be an active protection. In individuals under T1 and T2, retraction of the tentacles was observed with simultaneous inflation of the oral disc, which facilitates shedding of sediment^40^. *D. pertusum* showed a similar behaviour as *C. huinayensis* under suspended sediments: both species almost always showed fully extended tentacles in the control treatment, while in the high sediment treatment the polyps were often only half open to closed, which is associated with reduced growth in both cases^35^. Retracted tentacles and mucus release by *D.* *pertusum* were also reported by Allers et al.^32^ when corals were covered with anoxic sediments (see Table 2). The cold-water soft coral *Duva florida* contracted when exposed to 8 mg L^−1^ rough edged mine tailings and remained contracted for up to 6 h thereafter^41^. For the warm-water gorgonian *Subergorgia suberosa*, Tseng et al.^63^ reported lower polyp extension when exposed to sediment loads of 50, 150, 250 mg L^−1^. These results suggest that corals maintain polyp activity up to a certain sediment concentration and some species may even increase polyp activity for cleaning, but that excessive sediment stress leads to a reduction in polyp activity. However, the lower polyp expansion probably impairs prey capture and thus coral nutrition, which is reflected in the lower growth rate of the heavily sediment-stressed corals (T2).

The increasing sediment concentration also caused the tissue retraction (coenosarc) of the juvenile *C. huinayensis*, exposing parts of their skeleton. This indicates that these corals were already compromised by the 100-times increased sediment load, but they were still able to maintain their growth rate. The related *D. dianthus* recruits also have a thin tissue layer that completely covers their skeleton, whereas adult corals partly retract their tissue and expose parts of the skeleton naturally^64^. However, age-related retraction of the coenosarc can be ruled out in our experiment with *C. huinayensis*, as all corals used were similarly young and no coenosarc retraction was observed in any of the control animals. Some WWC can cyclically expand and retract their tissue, for instance when corals fall dry due to the tidal rhythm and retract their tissue as a “protective mechanism” against increased solar radiation^65,66^. In the present case, however, there is no such influence of tides, as the corals are permanently covered by water, and the retraction of the coenosarc thus most probably represents a stress response to the increased sediment exposure. Further, sediment particles can damage the tissue and additionally lead to tissue loss; in both cases, the loss of tissue negatively impacts coral performance, as the bare skeleton is no longer protected from colonization by for instance, microborers or bacteria, which can weaken the skeleton^36,64,67,68^. To what extent tissue retraction harms the juvenile corals or helps them to survive potential longer stress situations—with possibly lower energy expenditure—should be further investigated. In addition, it is important to find out to what extent *C.* *huinayensis* can recover from tissue loss and whether there is a point at which recovery can no longer take place. In the case of *D.* *pertusum*, a high tissue recovery potential was found under aquarium conditions in coral fragments that broke off during sampling^69^. After repeated exposure to sediment, the ability of *D.* *pertusum* to grow new coral tissue over the freed skeleton decreased (26%)^36^. When sedimentation stopped, juveniles are likely to recover and grow new tissue^8^, but this depends on the condition of the polyp^36^ and whether other stressors affect the animals^30^.

Corals can actively remove sediment by increasing cilia and tentacle activity, expanding their body and secreting mucus^8,30,32,36–38,40^. During the experiment, small clumps of sediment were observed on the bottom of the beakers of T1 and T2 and rarely small threads of mucus were detected emanating from the corals. In addition, one specimen (under T1) showed a mucus sheet (Fig. SM3), indicating that *C.* *huinayensis* produces mucus under stress conditions, which is not surprising as this is a typical stress reaction for corals^38^. However, this is the first time that mucus production has been documented in *C. huinayensis*. Mucus production as a clearing behaviour and stress response well known from WWC^8,30,38^ and CWC^32,36^. In general, corals trap sediment in mucus and repel it as an aggregate^38^, which is an energetically costly process^40^. Increasing mucus production due to stressors, such as sediment, therefore has a negative effect on the energy balance of the animals^59^.

Further, we detected extruded mesenterial filaments in some corals under T2. For WWCs, extruded mesenterial filaments have been reported as a defence mechanism^70,71^, and as a sign of stress^72^ and have been reported as a response to sedimentation^40^. The CWC *D. dianthus* showed evagination of its mesenteries under acidified conditions along with a decrease in skeletal growth^73^. Thus, the extruded mesenterial filaments of *C. huinayensis* under T2 are likely a stress sign (Fig. 4).

Furthermore, growth rate, polyp extension and tissue retraction reveal that those individuals placed in a sideways position are negatively affected. As sedimentation is a vertically oriented process^74^, sediment is less likely to remain on downward oriented individuals. There is also a significant correlation between the growth form of the corals and their sediment tolerance, with columnar, thin and branching coral species being the most tolerant^40^. This is because growth form can be a passive strategy against sediment, as some forms can withstand strong currents or provide more surface area exposed to the current so that sediment particles are simply washed away^40^. The CWC *C. huinayensis* occurs on vertical and downslope walls, and this position was imitated in this study. They rarely occur on sideways oriented substrate. Probably because they get covered and would get smothered under the high sediment load. The same applies for larval recruitment, which is why settlement is more successful on steep walls and overhangs, where sediment is harder to accumulate, abrasion avoided and therefore recruit survival increased^40,75^. High turbidity—which could result from road construction in Comau Fjord—may also affect successful settlement and lead to lower recruitment, as sediment can clog larval feeding and swimming structures and clogged larvae may sink to the ground^34^. As natural survival rates for recruits are already low, this reduces success^9,30,76^.

The impact and coral response to sediment load in the natural environment is influenced by additional factors, such as currents^45^ and food supply^51,52,77,78^. Furthermore, the sediment-induced tissue regression suggests a deterioration or increasing influence on the coenosarc cover of the animals under T1 and T2 over time. Therefore, a longer duration of the exposure may exacerbate the severe effects on the physiological performance of the corals and ultimately lead to increased mortality^40,77^. This is particularly important in the case of continuous sedimentation stress from aquaculture or from a busy road. Aquaculture increases sedimentation loads^19^, which in combination with other stressors (e.g. eutrophication, hypoxic or anoxic conditions, harmful algal blooms^15,20^) triggered by aquaculture can lead to lower thresholds and must not be ignored. Further, recurrent stress events can also have devastating effects, especially if the animals have not yet had time to recover. Local effects of sedimentation are increasingly joined by anthropogenic hazards (e.g. global warming, ocean acidification) that interact as multiple-stressors and can lead to cumulative effects with significant impacts^79^.

## Conclusion

Anthropogenic impacts increase sediment input to marine ecosystems, posing a threat to habitat forming CWCs. Juveniles, which are essential for the conservation of the species, are particularly at risk. The 12-week *ex-situ* experiment showed that juveniles of *C. huinayensis* can cope to a certain extent with naturally elevated sediment loads. However, sublethal effects such as reduced growth, respiration, and activity as well as tissue decline, indicate that the animals use their energy for the most important survival functions. As ocean warming and associated ocean acidification are expected to increase in the future, experiments with multiple stressors are essential to gain better insight into the effects and future evolution of the Chilean CWC habitats. At some sites of Comau Fjord, the documented natural seawater pH reaches values predicted for the oceans by the end of the century^80^ and rising sediment loads pose a major risk. Considering the results of this study and recognition that a severe reduction or even disappearance of a framework-forming species can have strong impacts on complex ecosystems^12,16,81^, management plans need to consider the deleterious effects of increased sedimentation on vulnerable CWC.

## Materials and methods

The 12-week sediment exposure experiment (19.01.2021–16.04.2021) included two treatments with different sediment concentrations and a control. This time period was chosen accordingly, as a stress experiment with the same species showed a delay in the stress response in terms of calcification rate; after 12 weeks, differences were statistically detectable^51^. Twenty juvenile specimens of the CWC *C.* *huinayensis* were used for each treatment, ten of which oriented sideways and ten downwards, corresponding to two natural growth directions. Growth was measured as calyx diameter and mass increase, further response variables measured were polyp behaviour, tissue retraction and respiration rates.

### Sediment sampling and processing

The top centimetre of Wadden Sea sediment was collected at Spieka-Neufeld (Germany, 53° 47′ 25.3′′ N, 8° 32′ 49.0′′ E). After sieving, the grain size fraction 63–125 µm (F1) and ≤ 63 µm (F2) were allowed to settle (two days) and excess water was decanted. The two fractions were mixed (3:7, F1:F2) to match the grain size distribution determined from a CWC aggregation in Chilean Comau Fjord^82^. To remove the organic matter (OM), the sediment was lyophilised, boiled in H_2_O_2_ (35% stabilized) until the reaction was completed, and washed with Milli-Q-Water (MQ). After sedimentation (five days), excess water was discarded. Washing and sedimentation was repeated three times to completely remove H_2_O_2_. The granulometry of the sediment mixture was analysed using a Beckman Coulter Laser Diffraction Particle Size Analyzer LS 13320 at MARUM, University of Bremen (for details see Supp. Mat. 4).

Three sediment concentrations were prepared in relation to the natural turbidity in Comau Fjord, which varies between 0.595 and 2.524 NTU below 18 m water depth^83^. For the natural sediment concentration (C), 4.2 µL of the moist sediment, corresponding to 1.6 mg sediment dry mass [DM], was mixed with one litre seawater (see also Verse^84^), which resulted in a turbidity of 1.5 ± 0.59 NTU. Treatment 1 (T1) and Treatment 2 (T2) consisted of 100-fold (420 µL **≙** 160 mg sediment DM L^−1^) and 1000-fold (4200 µL **≙** 1600 mg sediment DM L^−1^) the natural sediment concentration (C), respectively (Tab. SM5). For each treatment, working solutions were prepared by diluting the sediment mixture in Milli-Q water (C 1:499, T1 1:4, T2 undiluted) and stored at 5 °C until use.

### Experimental set-up

**Figure 5:**
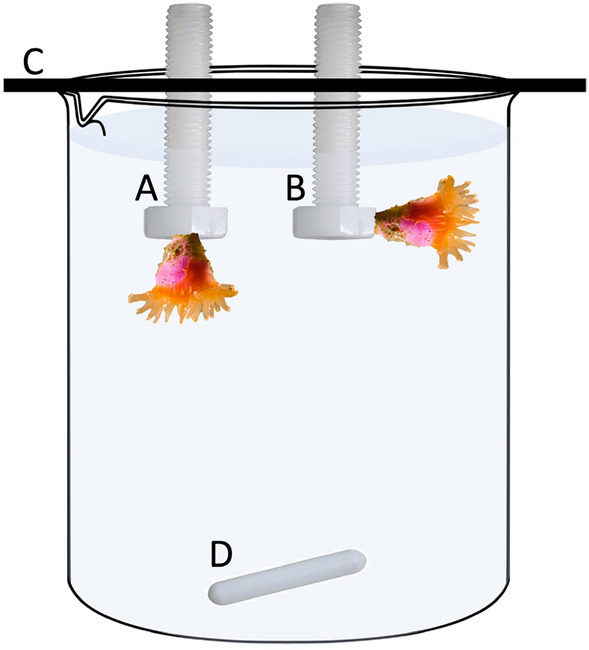
Beaker with test corals glued on polyamide screws facing downwards (**A**) and sideways (**B**). The screws were screwed into a plexiglass holder placed on the beaker (**C**). A stir bar (**D**) driven by a magnetic stirrer (200 rpm) under the beaker provided moderate flow. Schematic illustration.

The juvenile corals were offspring of *C.* *huinayensis* kept in the aquaria of the Alfred-Wegener-Institut Helmholtz-Zentrum für Polar- und Meeresforschung after being collected in Comau Fjord in 2015 (see Laudien et al.^85^). Specimens of similar size (calyx diameter 1.5 to 3.2 mm) were removed from the aquarium walls with a razor blade and glued with Super Glue Gel (i) sideways or (ii) downwards onto numbered polyamide screws (Fig. 5). Corals were acclimatized in a separate aquarium circuit for 1 week. Four controls were used per treatment: one screw without coral and three screws with bare coral skeletons. During the acclimation period and experiment, corals were fed 24-h-old live *Artemia persimilis*-nauplii (hatched from 7.76 mg cysts L^−1^ beaker) and liquid coral food (6 µL L^−1^; MIN S, Fauna Marin, Germany) three times per week for a period of 4 h, followed by a water exchange.

**Figure 6:**
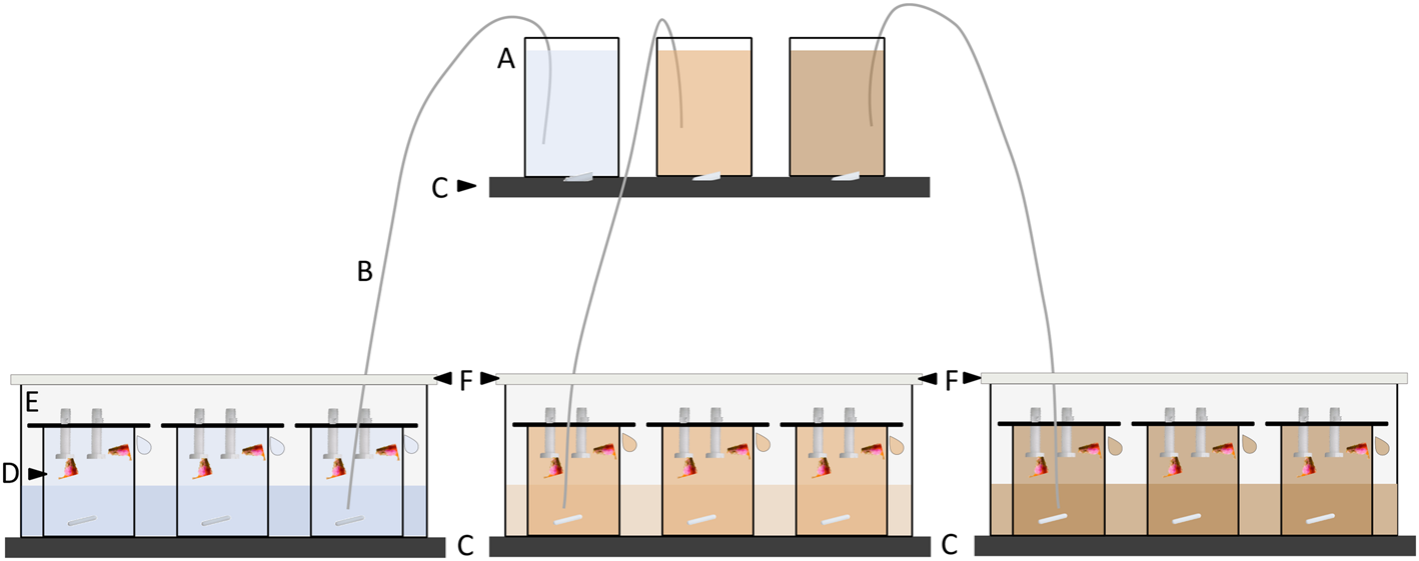
Schematic illustration of experimental set-up. (**A**) glass containers with three different sediment suspension, (**B**) tubing for rinsing the suspension into beakers, (**C**) multipoint stirrer, (**D**) beaker filled with sediment suspension and plexiglass holder on top with a coral glued to a screw, (**E**) overflow collection trays and water bath, (**F**) Styrofoam plates to slow down evaporation and keep the temperature in the beaker stable.

The three different sediment suspensions were prepared in 650 mL-glass containers. These were filled with filtered (pore size 0.2 µm) artificial seawater with the same characteristics (i.e. temperature, salinity, nutrient and oxygen concentration) as the water from the sampling site of the parent corals^85^. The thawed stock sediment solutions were homogenised with a vortex and the treatment solution prepared. The glass containers were placed on a multipoint stirrer at 350 rpm with a stir bar to keep the sediment in suspension (Fig. 6).

At the beginning of the experiment, ten 340 mL-beakers per treatment were filled each by gravity from a 650 mL-glass container via a thin tube with the respective sediment solution and positioned in a water bath (11 °C) on the multipoint stirrer (200 rpm) in a dark temperature-controlled room (Fig. 6). Water temperature, salinity, oxygen and nutrient contents were monitored three times a week throughout the experimental period. The screws of the acclimatised corals were carefully cleaned of fouling organisms and particles with a toothbrush and then exposed to the treatments. Water exchange (> 60% of the water volume was renewed) took place three times a week in the same way as during the first filling. Response variables were measured at the beginning of the experiment and after 6 and 12 weeks.

### Physical and chemical environmental conditions

#### Turbidity

Turbidity was measured daily or immediately before feeding and after a water exchange (two times per day) throughout the duration of the experiment. From each of five beakers per treatment, 2 mL incubation water were collected with a syringe, pooled (10 mL in total) and turbidity was measured three times with a turbidimeter (2100Qis, Hach Lange): Turbidity of the incubation water was significantly different between treatments (Kruskal–Wallis: *p* < 0.001). However, turbidity varied over time, with highest values (C: 1.7 ± 0.45 Formazine Nephelometric Units (FNU); T1: 59.9 ± 19.68 FNU; T2: 629.4 ± 206.31 FNU) measured immediately after water exchanges and the lowest (C: 1.1 ± 0.6 FNU; T1: 12.3 ± 3.41 FNU; T2: 276.9 ± 113.45 FNU) measured 3 days later just before another water change (Supp. Mat. 7). The sediment concentration thus decreased between water changes in each treatment and corals were partly exposed to lower concentrations. However, the intervals between water change were small and the fluctuation reflects natural variations.

#### Granulometry

Grain sizes of the sediment suspension in the beakers was measured with a Beckman Coulter Laser Diffraction Particle Size Analyzer LS 13320 at MARUM, University of Bremen (Supp. Mat. 4). Sediment samples collected from the beakers of T1 and T2 showed no differences in grain size distribution (Kruskal–Wallis test: *p* > 0.05). The sediment consisted mainly of a high proportion of silt (grain size: > 3.9 µm to < 63 µm, 69.42 ± 5.65 vol.%) and clay (grain size < 3.9 µm, 29.94 ± 5.62 vol.%), while sand-sized particles (> 63 µm) were rare (< 1 vol.%) (Fig. SM4).

#### Physical and chemical water properties

Nitrate, nitrite, ammonium and phosphate were analysed three times per week throughout the experimental period from two bulk samples of five beakers each from one treatment. Precision nutrient measurements were performed weekly using photometer test sets (NOVA 60, Spectoquant® tests for ammonium 114752, nitrite 114776; phosphate 114848; nitrate 114942; ammonium 114752, Merck, Germany). Test kits were used twice a week to check for nutrient values in between (JBL PROAQUATEST for nitrite, nitrate, phosphate, ammonium; Salifert Phosphate PO_4_ Profi Test). Total alkalinity (TA) was measured weekly in triplicate using potentiometric titration (TitroLine alpha plus)^86^ and North Sea water as standard, which was corrected against Dickson standard seawater (Dickson, Scripps Institution of Oceanography). From this, aragonite values were calculated. Temperature, salinity and dissolved oxygen concentration were assessed with a temperature sensor (WTW ama-digit) three times per week before water changes. Incubation water conditions were similar with a temperature of 11.08 ± 0.68 °C, salinity of 32.64 ± 0.48, pH of 8.12 ± 0.03 and oxygen saturation of 97.88 ± 2.77% (Supp. Mat. 7). There were no significant differences in nitrate, nitrite or ammonium concentration (Kruskal–Wallis: *p* > 0.05), while phosphate concentration increased with the amount of sediment and showed significant differences between all three treatments (Kruskal–Wallis + post-hoc Dunn-Bonferroni: *p* < 0.001). However, an experiment showed, that the increased phosphate concentration in T2 was not causing the significant reduced growth rate (see Supp. Mat. 2). The aragonite saturation ${(\Omega }_{arag})$ showed no significant difference between treatments (ANOVA, *p* > 0.05) (Supp. Mat. 7).

### Response variables

#### Growth

The mass changes of the corals were determined using the buoyant weight method^87^. The screw with the polyp was carefully cleaned with a toothbrush, weighed before (week 0), between (week 6) and at the end (week 12) of the experiment. Each screw with the polyp attached was weighed three times, then the values were averaged and the skeletal mass calculated according to Jokiel et al.^87^. The skeletal density of *C.* *huinayensis* was calculated after Davies^88^. Two specimens (one sideways oriented from T1 and one downward oriented from T2) that fell off during the experiment and had to be re-glued were excluded from further mass measurements, as in principle an acclimatisation period must be maintained so that the animals do not initially deliver biased growth rates because they are stressed. In addition, the polymer from which the screw is made has the capacity to absorb water, which replaces the gas and thus will get heavier in the first 2 weeks^97^. Furthermore, the glue may show mass differences when settling^98^.

The variation in calyx diameter was determined from photographs taken in zenith view under a stereomicroscope (LEICA MZ 16, Leica Microsystems) with the software Leica Acquire 3.4.1. Build 9072 (Leica Microsystems). The calyx diameter was measured with the software ImageJ (Version 1.53) and the diameter calculated from the near round calyx of *C. huinayensis*^45^.

Mass and calyx variations were determined as percent increase per day ($G$) as follows:

1 $$G\left( {\% d^{ - 1} } \right) = \frac{{\left( {D_{Te} - D_{Ts} } \right)}}{{\left( {D_{Ts} \times \left( {Te - Ts} \right)} \right)}} \times 100$$

where ${D}_{Ts}$ and ${D}_{Te}$ are the mass (mg) and the calyx diameter (mm) at the start ($Ts$) and at the end ($Te$) of the measurements, respectively, $Te$ − $Ts$ is the duration of the experiment in days (d). Negative values were set to zero if they were within the measurement error (< 1%).

#### Polyp expansion

**Figure 7:**
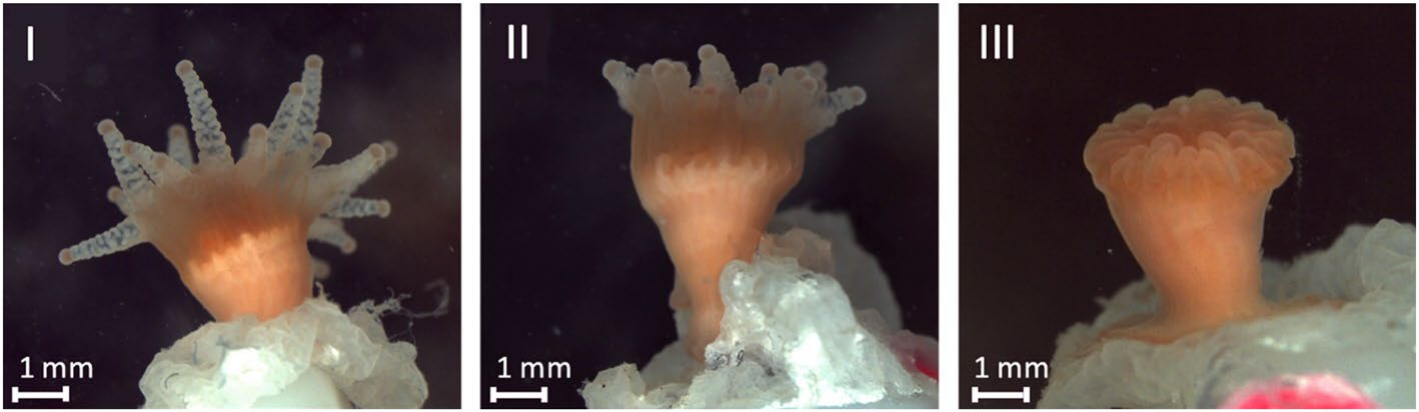
Photographs of juvenile *C. huinayensis* exemplifying the defined polyp activity categories. I: fully expanded, II: partly expanded, III: fully retracted polyp.

Polyp expansion was documented four times per week at the same time of the day per individual from one week after the start of the experiment. We defined three categories of polyp activity: I = polyp fully expanded, II = polyp partly expanded, and III = polyp fully retracted (Fig. 7). The percentage of animals in each category and for each treatment was then calculated over the duration of the experiment.

#### Tissue retraction

Tissue retraction of juvenile *C. huinayensis* was documented using the stereomicroscope and Leica Acquire software. Observations were conducted 6 and 12 weeks after the start of the experiment. The percentage of individuals displaying partial to full or no tissue retraction was recorded per treatment.

#### Oxygen consumption

Respiration measurements were conducted once on ten haphazardly taken individuals (five sideways and five downwards facing) per treatment 1 week after the end of the experiment to avoid handling effects of the last measurement (after 13 weeks of incubation). The corals were last fed 24 h before the incubation to exclude potential effects due to digestion^57^. Measurements were also conducted in two control beakers, which included just a screw with glue.

To measure respiration under different sediment loads (C, T1 and T2), custom made closed 20 cm^3^ respiration chambers were used. To ensure that the sediment suspension was homogeneous, chambers were filled with the continuously mixed sediment suspension from the glass containers as described previously. The screw with the glued-on coral was attached to the stopper of a glass chamber, then the chamber was closed without air bubbles. Salinity and pH were measured (see above) at the beginning of the measurements, and turbidity was measured after the incubations. Chambers were placed in the dark in a water bath (11 °C) on a multipoint stirrer. Water circulation and sediment suspension were maintained in the chambers using magnetic stir bars at 180 rpm. Each chamber was equipped with an optical oxygen sensor spot (OXSP5, PyroScience) and fibre-optical sensors (2 m, SPFIB-BARE, PyroScience) connected to a multi-channel oxygen meter (FireSting^®^-O_2_, PyroScience). Temperature sensors (Pt100 TSUB21, PyroScience) were also connected to each transmitter. Prior to the measurements, the oxygen sensors were calibrated with O_2_-free and O_2_-saturated artificial seawater. Throughout the 24 h incubation period, oxygen saturation was measured every ten seconds and recorded using the PyroScience Workbench software (V1.2.0.1359; PyroScience). Values were calculated in µmol L^−1^ and mg L^−1^ using the Oxygen Calculation Tool (PyroScience) for MS Excel, considering salinity, temperature, and calibration settings. Oxygen concentrations were grouped at three-hour intervals to check whether respiration was linear over time (R^2^ > 0.95), and then metabolic rates were calculated with the r package rMR (function MR.loops^89^). Subsequently, mean mass-specific (AFDM) respiration rates, where R is the respiration rate, were adjusted for the volume of the respiration chambers (V_Inc_, mL) and bacterial background respiration (R_BG_) was calculated according to Eq. (2) and expressed as respiration rate per day (R_d_). From this, the average respiration rate was calculated as mean value for all chambers per treatment.

2 $$R_{d} = \frac{{\left( {R - R_{BG} } \right) \times \frac{{V_{Inc} }}{1000} }}{AFDM} \times 24$$

Immediately after the respiration measurement, the coral was snap-frozen and stored at − 80 °C to later determine AFDM. Therefore, corals were dried individually in pre-combusted aluminium trays at 40 °C for 24 h, weighed (precession 0.1 mg, CPA225D-0CE, Sartorius), combusted in a muffle furnace at 500 °C for 24 h, and then weighed again. Subsequently, ash mass was subtracted from dry mass.

### Statistical analyses

Statistical analyses and graphs were performed with RStudio^90^. All data were tested for normal distribution using Shapiro–Wilk test^91^ and for variance homogeneity using Levene’s test^92^. For the respiration data, potential statistically significant differences between corals of the treatments were determined using analysis of variance (ANOVA) and identified between groups using Tukey’s honestly significant difference (HSD)^93^. Data, which were not normally distributed after transformation (logarithmic, Box-Cox) were analysed with conservative tests: a Kruskal–Wallis test^94^ and the post-hoc Dunn-Bonferroni test^95^; these analyses were performed to indicate potential differences between treatments for the measured response variables: growth (buoyant weight, calyx growth), polyp activity and tissue retraction. For statistical comparisons between response variables in different treatments and their orientation of the non-parametric data (buoyant weight, calyx growth, polyp activity, tissue retraction) the Scheirer-Ray-Hare test (SRH-test)^96^ was performed. All results are expressed as mean ± SD.

### Supplementary Information

Supplementary Information 1.

## Data Availability

Photographs of the calyx and the lateral side of the individuals, as well as all data presented in this paper on mass, calyx increase, turbidity, grain size of sediment, pH, temperature, salinity, oxygen, and nutrients can be assessed at the World Data Centre PANGAEA: https://doi.pangaea.de/10.1594/PANGAEA.941516. The datasets used and/or analysed during the current study are available from the corresponding author on reasonable request.
