# Supplementary material for: Ecophysiological and behavioural response of juveniles of the Chilean cold-water coral Caryophyllia (Caryophyllia) huinayensis to increasing sediment loads
Source: Sci Rep. 2023 Dec 6;13:21538. doi: 10.1038/s41598-023-47116-6 (PMC10700329; doi:10.1038/s41598-023-47116-6)
Supplement: Supplementary file 1 — Supplementary Information 1. [file 41598_2023_47116_MOESM1_ESM.pdf]

Supplementary material

Supplementary material 1:
Growth rates for juvenile *C. huinayensis* under C and T1 conditions are intermediate between the *in-situ* growth rates measured for two other stony coral species sharing habitat in Comau Fjord: *D. dianthus* (0.25 ± 0.18 % d^{-1})[1] and *T. endesa* (0.03 ± 0.02 % d^{-1})[2]. However, *ex situ* growth rates measured *for D. dianthus* individuals from Comau Fjord and the Mediterranean Sea are lower (0.09 ± 0.08, 0.06 ± 0.03 % d^{-1}, respectively)[1,3]. The higher *in situ* growth rates may be due to an overestimation from the 2-week measurement conducted in summer by Jantzen et al.[1], as the corals may show seasonal growth variation due to varying food availability[4–6], while *ex situ* growth rates were measured under controlled conditions, regulating factors that influence growth (e.g. food availability, temperature)[7]. A factor influencing coral growth rates is the ontogeny of the animals, as growth rate varies with age[4,8–12]. As coral polyps age, their growth rate decreases[9,12], while stress factors such as low pH affects the growth of young polyps more than that of older polyps[9]. The comparatively high growth rates of *C. huinayensis* under C and T1 conditions are due to their young age and thus stronger growth. In mature corals, further reproduction may play a role as energy is used for germ cell production[13] (Heran et al., submitted). That growth rates vary greatly between CWC species is also indicated by the branching *M. oculata,* which has *ex situ* growth rates of 0.11 ± 0.04 % d^{-1} over two years[3], while the branching *D. pertusum* has *in situ* and *ex situ* growth rate of only 0.01 and 0.02 ± 0.01 % d^{-1}, respectively[3,14]. Nevertheless, the measured growth of corals under T2 is lower than that of other *in* or *ex situ* grown CWCs (Tab. SM1). Thus, it appears that the high sediment load significantly affects growth rates and leads to a marked decline.

Species	Location	Increase (% d^{-1})	Time	Temp (°C)	Exp. Setup	Reference
C. huinayensis (juvenile)	Comau Fjord	0.18 ± 0.12	12 w	11	*ex-situ*	This study
D. dianthus	Comau Fjord	0.25 ± 0.18	2 w	12	*in-situ*	Jantzen et al.[1]
D. dianthus	Comau Fjord	0.09 ± 0.08	2 w	12	*ex-situ*	Jantzen et al.[1]
D. dianthus	Mediterranean Sea	0.06 ± 0.03	8 m	12	*ex-situ*	Orejas et al.[3]
T. endesa	Comau Fjord	0.03 ± 0.02	1 y	12	*in-situ*	Rossbach et al.[2]
D. pertusum	North Atlantic	0.01 ± 0.01	1 y	8	*in-situ*	Büscher et al.[14]
D. pertusum	Mediterranean Sea	0.02 ± 0.01	1 y	12	*ex-situ*	Orejas et al.[3]
M. oculata	Mediterranean Sea	0.11 ± 0.04	2 y	12	*ex-situ*	Orejas et al.[3]

Table SM1. Growth rates of CWCs determined by the buoyant weight method; y = year, m = month, w = weeks

Supplementary material 2:
To rule out the possibility that elevated phosphate levels resulting from increased sediment in T2 would not affect growth, corals were exposed to elevated phosphate levels without additional turbidity in another experiment. For this, the sediment concentration of T2 was prepared in 650 ml filtered artificial seawater and kept in suspension on a magnetic stirrer in a temperature-controlled room for two days. The water was then filtered (cellulose membrane filter). Analogous to the long-term experiment, corals were placed facing downwards in this water next to the control treatment under a light current for 36 days to subsequently determine the mass difference. Corals exposed to increased (PO_4^{3-}) had a significant higher growth rate than individuals of T2 (Kruskal-Wallis test + post-hoc Donn-Bonferroni: $p < 0.05$).

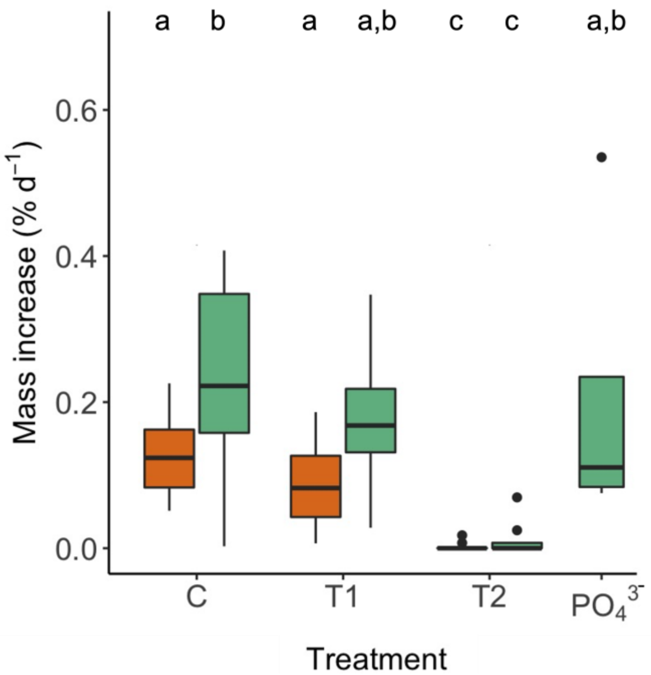

Figure SM2. Variation in mass of juvenile *C. huinayensis* under C, T1, T2 conditions in % per day and to increased phosphate values (PO_4^{3-}). Orange boxes display growth of sideways oriented corals and green boxes the downward oriented ones. The box represents the interquartile range of replicates with median (black horizontal line), standard deviations (whiskers) and outliers (black dots). The significance levels between treatments are indicated as letters, with different letters representing significant differences.

Supplementary material 3:

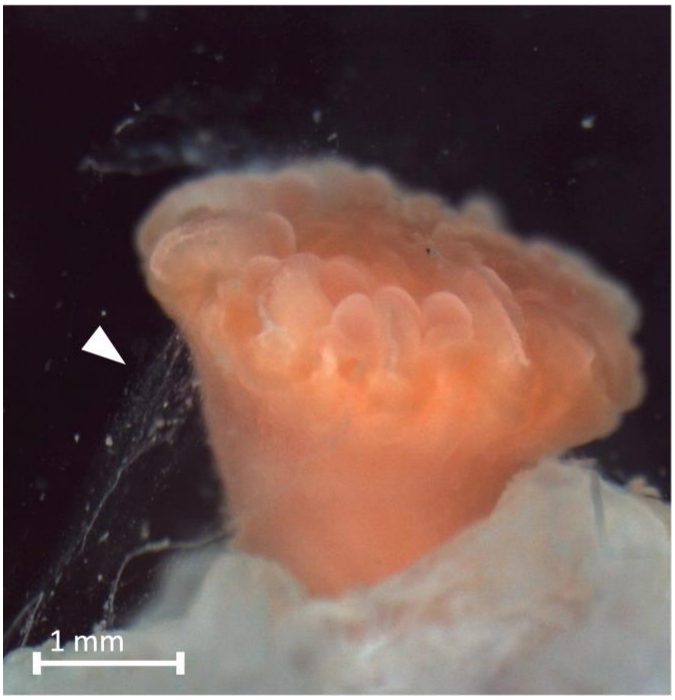

Figure SM3. Mucus (marked with arrow) from a juvenile *C. huinayensis* kept under T1 for 6 weeks. Scale bar 1 mm.

Supplementary material 4:

Granulometry
To characterise the sediment in the natural habitat of *C. huinayensis*, the grain size distribution of sediment collected from 17 sediment traps (20.01.2014 – 16.02.2014) positioned directly in their natural habitat in Comau Fjord, Chile, were analysed by Gottschlich[15, 16]. Grain size measurements were performed in the Particle-Size Laboratory at AWI Sylt with a Quantachrome Cilas 1180 Particle Analyzer that provides grain size distributions from 0.04 to 2500 µm divided into 161 size classes. Prior to the measurement, the organic carbon was removed by a sediment pre-treatment with H_2O_2 until the reaction stopped.

For the present experiment, sediment was collected from the Wadden Sea by Spieker-Neufeld (Germany) in October 2020 and February 2021. Before use in the experiment, the sediment was sieved into fractions that were mixed proportionally to match the size range of the sediments in Comau Fjord (see main text for details). Organic carbon was then removed with H_2O_2 until the reaction stopped and the sediment was washed for neutralisation. Grain size measurements of the sediment used in the experiments were performed before, during and after the experiment in the Particle-Size Laboratory at MARUM, University of Bremen with a Beckman Coulter Laser Diffraction Particle Size Analyzer LS 13320 that provides grain size distributions in the size range from 0.04 to 2000 µm, divided into 116 size classes.

The measurements showed that the Wadden Sea sediments were generally in the same grain size range between 0.24 and 250 µm as the natural sediment in Comau Fjord. However, the relative size distribution deviated considerably. Comau Fjord sediments exhibited a bimodal distribution with a coarse mode between 63 and 125 µm and a fine mode between 31 and 15.6 µm. The Wadden Sea sediments showed a polymodal distribution with two major modes: a coarse mode between 63 and 125 µm, not very prominent in the sediment collected in February, and a relatively broad fine mode between 3.9 and 15.6 µm. As the sediment in sediment traps tends to reflect sediment reaching the site (input-dependent sediment fraction), but not the sediment that remains in suspension for a longer period of time and impacts the corals, which depends more on the turbulence of the bottom water (bottom-current dependent sediment fraction), the grain size distribution of the Wadden Sea sediments is considered to reflect the natural sediment in Comau Fjord well-enough to be used for the experiments performed.

Measurements of the grain size distribution of the sediment suspension during and after the experiment (T1 and T2) showed a predominance of the fine mode and a nearly lack of the coarse mode compared to the input Wadden Sea sediments. This change in grain size distribution likely resulted from the settling of the coarse fraction within the beakers. Accordingly, the turbulence generated by the magnetic stirrer was not strong enough to keep the coarse fraction in suspension. Comparing the grain size distributions, as well as the modes of the two Wadden Sea source sediments (October and February) showed only minor deviations during the experiment. This suggests that a potential influence of the different grain size distributions of the source sediments is rather unlikely and can be ignored for the interpretation of the results of the experiment.

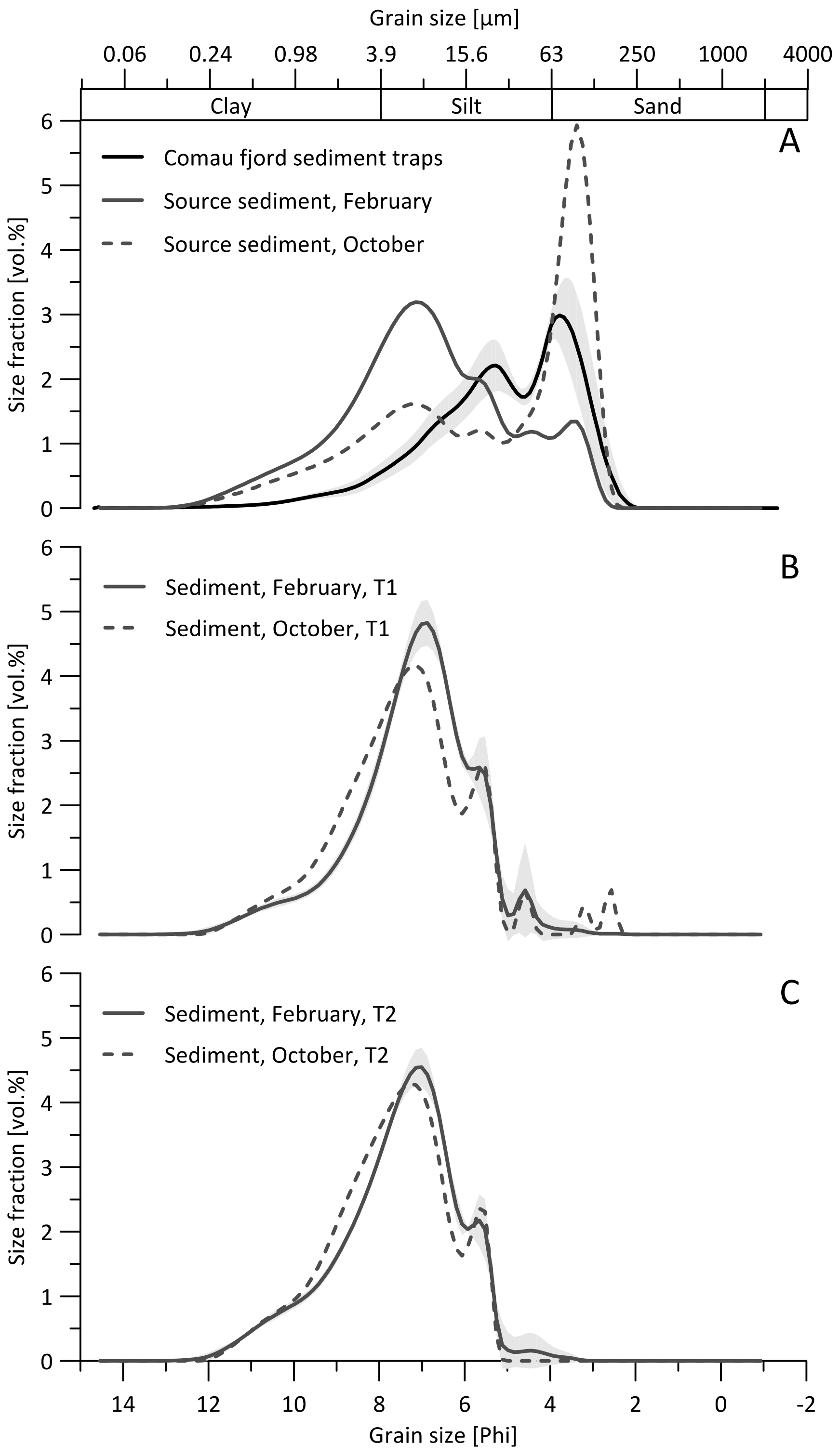

Figure SM4. (A) Grain size distribution of sediment collected in sediment traps (number of replicates = 17) from Comau Fjord, Chile, and the Wadden Sea source sediments used in the herein performed experiment (February: number of replicates = 3; October: number of replicates = 1). **(B)** Grain size distribution of suspended sediment during the experiment (at T1) of both Wadden Sea source sediments (February: number of replicates = 3; October: number of replicates = 1). **(C)** Grain size distribution of suspended sediment after the experiment (at T2) of both Wadden Sea source sediments (February: number of replicates = 3; October: number of replicates = 1). Grey area indicates the standard deviation of the replicate measurements. Note: standard deviation of the replicate measurements for the source sediment February was so small that it is not visible.

Supplementary material 5:

	C	T1	T2	Ambient below 18 m water depth
Sediment concentration (ml l^{-1})	0.0042	0.42	4.2	
Equivalent dry sediment (mg l^{-1})	1.6	160	1600	
Turbidity FNU	1.5 ± 0.59	30.4 ± 26.25	425.1 ± 228.82	0.76 ± 0.07 (min 0.60, max 2.52)

Table SM5 Sediment concentrations and corresponding turbidity used for the sediment-stress experiment.

Supplementary material 6:

Response variable	Orien-tation	C	T1	T2	Statistical test	Vari-able	Df	H-value	F-value	chi-squared	p-value	Residuals
Mass increase (% d^{-1})	A	0.18 ± 0.115	0.132 ± 0.089	0.007 ± 0.017	Scheirer-Ray-Hare	T	3	36.842			**<0.001**	55
						O	1	3.266			**0.046**	
						T:O	2	0.903			0.637	
	S	0.12 ± 0.056	0.09 ± 0.058	0.002 ± 0.006	Kruskal-Wallis	**T**	2			19.132	**$7.007 e^{-5}$**	
	D	0.24 ± 0.133	0.17 ± 0.098	0.01 ± 0.023	Kruskal-Wallis	**T**	2			16.953	**< 0.001**	
Calyx diameter (% d^{-1})	A	0.337 ± 0.124	0.319± 0.154	0.017 ± 0.029	Scheirer-Ray-Hare	T	2	38.767			**<0.001**	53
						O	1	0.258			0.611	
						T:O		0.247			0.884	
	S	0.34 ± 0.138	0.29 ± 0.154	0.01 ± 0.021	Kruskal-Wallis	T	2			19.578	**$5.606 e^{-5}$**	
	D	0.35 ± 0.114	0.34 ± 0.152	0.03 ± 0.033	Kruskal-Wallis	T	2			19.12	**$7.048 e^{-5}$**	
Oxygen consumption (*µg O2 mg AFDM^{-1} d^{-1}*)	A	11.3 ± 3.8	7.5 ± 2.4	3.9 ± 1.9	ANOVA	T	2		17.82		**$1.16 e^{-5}$**	27
					two-ANOVA	O	1		0.748		0.396	24
						T:O			0.630		0.541	
	S	10.12 ± 3.922	7.23 ± 2.224	4.06 ± 2.367	ANOVA	T	2		5.306		**0.0223**	12
	D	12.57 ± 3.54	7.84 ± 2.794	3.7 ± 1.549	ANOVA	T	2		13		**< 0.001**	12
Polyp expansion Category I (%)	A	85.25 ± 12.22	85.73 ± 19.57	5.95 ± 12.04	Scheirer-Ray-Hare	T	2	121.426			**< 0.001**	240
						O	1	2.859			0.091	
						T:O	2	6116			**0.047**	
	S	86.25 ± 13.34	79.76 ± 20.8	2.14 ±4.70	Kruskal-Wallis	T	2			84.06	**< $2.2 e^{-16}$**	
	D	84.25 ± 11.07	91.71 ± 16.42	9.76 ±15.54	Kruskal-Wallis	T	2			91.348	**< $2.2 e^{-16}$**	
	A	13.25 ± 11.34	12.56 ± 19.42	59.64 ± 28.93		T	2	121.426			**< 0.001**	240

Response variable	Orien-tation	C	T1	T2	Statistical test	Vari-able	Df	H-value	F-value	chi-squared	p-value	Residuals
Polyp expansion Category II (%)					Scheirer-Ray-Hare	O	1	0.118			0.732	
						T:O	2	12.712			**0.00174**	
	S	12.25 ± 12.71	17.56 ± 21.19	45 ± 24.12	Kruskal-Wallis	T	2			46.345	**8.637 e^{-11}**	
	D	14.25 ± 9.84	7.56 ± 16.25	74.29 ± 25.96	Kruskal-Wallis	T	2			79.965	**< 2.2 e^{-16}**	
Polyp expansion Category III (%)	A	1.25 ± 4.02	1.1 ± 3.85	32.38 ± 26.28	Scheirer-Ray-Hare	T	2	142.54			**<0.001**	240
						O	1	7.672			**0.0056**	
						T:O	2	13.610			**0.0011**	
	S	1 ± 3.79	1.46 ± 4.22	49.29 ± 23.41	Kruskal-Wallis	T	2			97.479	**< 2.2 e^{-16}**	
	D	1.5 ± 4.27	0.73 ± 3.46	15.48 ± 16.26	Kruskal-Wallis	T	2			48.847	**2.471 e^{-11}**	

Table SM6. Mean response variables (± SD) divided into the different orientations (A: Overall, S: Sideways, D: Downwards) for the three sediment concentrations (C, T1, T2) and results of the statistical analysis indicating the variables tested (T = Treatment, O = Orientation). Significance differences marked in bold. Mass increase: Overall C, T2 n = 20, T1 = 19, sideways n = downwards n = Calyx diameter and polyp expansion: overall n = 20, sideways/downwards n = 10; Oxygen consumption: overall n = 10, sidewasy/downwards n=5.

Supplementary material 7:

Sediment concentration	**C**	**T1**	**T2**	**Statistical test**	**Df**	**chi-squared**	**F-value**	**p-value**	**Residuals**
Turbidity (FNU)	1.5 ± 0.59	30.4 ± 26.25	425.1 ± 228.82	Kruskal Wallis	2	210.36		**< 2.2e^{-16}**	
Temp. (°C)	10.89 ± 0.576	11.06 ± 0.73	11.3 ± 0.68	Kruskal- Wallis	2	10.078		**0.006**	
Salinity	32.79 ± 0.48	32.78 ± 0.46	32.34 ± 0.39	Kruskal- Wallis	2	21.858		**1.79 e^{-5}**	
pH	8.115 ± 0.035	8.124 ± 0.024	8.116 ± 0.039	ANOVA	2		0.549	0.579	99
Oxygen (mg l^{-1})	8.815 ± 0.227	8.838 ± 0.235	9.056 ± 1.588	Kruskal- Wallis	2	0.859		0.651	
NH_4 (mg l^{-1})	0.16 ± 0.074	0.13 ± 0.048	0.14 ± 0.048	Kruskal- Wallis	2	0.826		0.662	
NO^{2-} (mg l^{-1})	1.55 ± 0.005	2.51 ± 0.008	2.05 ± 0.015	Kruskal- Wallis	2	3.107		0.212	
NO^{3-} (mg l^{-1})	1.42 ± 1.551	1.59 ± 2.511	1.39 ± 2.049	Kruskal- Wallis	2	1.431		0.489	
PO_4^{3-} (mg l^{-1})	0.18 ± 0.06	0.33 ± 0.062	0.83 ± 0.145	Kruskal- Wallis	2	50.368		**0.156 e^{-10}**	
TA (µmol kg^{-1})	3039.64 ± 76.66	3040.17 ± 77.69	2819.81 ± 85.53	ANOVA	2		27.71	**1.52 e$^{-0.7}$**	30
Ω_{arag}	3.35 ± 0.57	3.46 ± 0.5	3.17 ± 0.43	ANOVA	2		0.963	0.393	30

Table SM7. Mean environmental conditions (± SD) within beakers with the different sediment concentrations (C, T1, T2) and the results of the statistical analysis. Significance differences marked in bold.

References

1. Jantzen, C. *et al.* In situ short-term growth rates of a cold-water coral. *Mar. Freshwater Res.* **64**, 631 (2013).
2. Rossbach, S., Rossbach, F. I., Häussermann, V., Försterra, G. & Laudien, J. *In situ* skeletal growth rates of the solitary cold-water coral *Tethocyathus endesa* from the Chilean Fjord Region. *Front. Mar. Sci.* **8**, 757702 (2021).
3. Orejas, C. *et al.* Long-term growth rates of four Mediterranean cold-water coral species maintained in aquaria. *Mar. Ecol. Prog. Ser.* **429**, 57–65 (2011).
4. Beck, K. K. *et al.* Ontogenetic differences in the response of the cold-water coral *Caryophyllia huinayensis* to ocean acidification, warming and food availability. *Sci. Total Environ.* **900**, 165565 (2023).
5. Beck, K. K. *et al.* Environmental stability and phenotypic plasticity benefit the cold-water coral *Desmophyllum dianthus* in an acidified fjord. *Commun Biol* **5**, 1–12 (2022).
6. Garcia-Herrera, N. *et al.* Seasonal and diel variations in the vertical distribution, composition, abundance and biomass of zooplankton in a deep Chilean Patagonian Fjord. *PeerJ* **10**, e12823 (2022).
7. Büscher, J. V., Form, A. U. & Riebesell, U. Interactive effects of ocean acidification and warming on growth, fitness and survival of the cold-water coral *Lophelia pertusa* under different food availabilities. *Front. Mar. Sci.* **4**, 101 (2017).
8. Lartaud, F. *et al.* A new approach for assessing cold-water coral growth *in situ* using fluorescent calcein staining. *Aquat. Living Resour.* **26**, 187–196 (2013).
9. Maier, C., Hegeman, J. & Weinbauer, M. G. Calcification of the cold-water coral *Lophelia pertusa* under ambient and reduced pH. *Biogeosciences* **6**, 1671–1680 (2009).
10. Martínez-Dios, A. *et al.* Effects of low pH and feeding on calcification rates of the cold-water coral *Desmophyllum dianthus*. *PeerJ* **8**, e8236 (2020).
11. Movilla, J. *et al.* Resistance of two Mediterranean cold-water coral species to low-pH conditions. *Water* **6**, 59–67 (2014).
12. Movilla, J. *et al.* Differential response of two Mediterranean cold-water coral species to ocean acidification. *Coral Reefs* **33**, 675–686 (2014).
13. Maier, S. R., Bannister, R. J., van Oevelen, D. & Kutti, T. Seasonal controls on the diet, metabolic activity, tissue reserves and growth of the cold-water coral *Lophelia pertusa*. *Coral Reefs* **39**, 173–187 (2020).
14. Büscher, J. V. *et al. In situ* growth and bioerosion rates of *Lophelia pertusa* in a Norwegian fjord and open shelf cold-water coral habitat. *PeerJ* **7**, e7586 (2019).
15. Gottschlich, S. Einfluss des Substrat-Neigungswinkels auf die Hartsubstratlebensgemeinschaft im Comau Fjord, Chile. *Bachelor Thesis (2014).*
16. Gottschlich, S., Laudien, J., Häussermann, V. & Försterra, G. Granulometry of sediments collected by differently inclined sediment traps in Comau Fjord. *2771 data points (2023) doi:10.1594/PANGAEA.955324.*
